# Microglia and glioblastoma heterocellular interplay sustains tumour growth and proliferation as an off‐target effect of radiotherapy

**DOI:** 10.1111/cpr.13606

**Published:** 2024-03-07

**Authors:** Cristiana Alberghina, Filippo Torrisi, Simona D'Aprile, Lucia Longhitano, Sebastiano Giallongo, Grazia Scandura, Giuliana Mannino, Stefania Mele, Maria Gabriella Sabini, Francesco P. Cammarata, Giorgio Russo, Ali S. Abdelhameed, Agata Zappalà, Debora Lo Furno, Rosario Giuffrida, Giovanni Li Volti, Daniele Tibullo, Nunzio Vicario, Rosalba Parenti

**Affiliations:** ^1^ Department of Biomedical and Biotechnological Sciences University of Catania Catania Italy; ^2^ Department of Medical, Surgical Sciences and Advanced Technologies “G.F. Ingrassia” University of Catania Catania Italy; ^3^ Department of Chemical, Biological, Pharmaceutical and Environmental Sciences University of Messina Messina Italy; ^4^ Medical Physics Unit Cannizzaro Hospital Catania Italy; ^5^ Laboratori Nazionali del Sud, INFN‐LNS, National Institute for Nuclear Physics Catania Italy; ^6^ Institute of Molecular Bioimaging and Physiology, National Research Council, IBFM‐CNR Cefalù Italy; ^7^ Department of Pharmaceutical Chemistry College of Pharmacy, King Saud University Riyadh Saudi Arabia; ^8^ Present address: Medicine and Surgery University of Enna “Kore” Enna Italy

## Abstract

Glioblastoma (GBM), a WHO grade IV glioma, is a malignant primary brain tumour for which combination of surgery, chemotherapy and radiotherapy is the first‐line approach despite adverse effects. Tumour microenvironment (TME) is characterized by an interplay of cells and soluble factors holding a critical role in neoplastic development. Significant pathophysiological changes have been found in GBM TME, such as glia activation and oxidative stress. Microglia play a crucial role in favouring GBM growth, representing target cells of immune escape mechanisms. Our study aims at analysing radiation‐induced effects in modulating intercellular communication and identifying the basis of protective mechanisms in radiation‐naïve GBM cells. Tumour cells were treated with conditioned media (CM) derived from 0, 2 or 15 Gy irradiated GBM cells or 0, 2 or 15 Gy irradiated human microglia. We demonstrated that irradiated microglia promote an increase of GBM cell lines proliferation through paracrine signalling. On the contrary, irradiated GBM‐derived CM affect viability, triggering cell death mechanisms. In addition, we investigated whether these processes involve mitochondrial mass, fitness and oxidative phosphorylation and how GBM cells respond at these induced alterations. Our study suggests that off‐target radiotherapy modulates microglia to support GBM proliferation and induce metabolic modifications.

## Graphical abstract

Radiotherapy‐induced effects, especially in whole brain irradiation, involve both tumour and surrounding healthy cells. Irradiated tumour and microglia cells create a complex network of intercellular communication. Our data reveal that off‐target irradiated microglia increase GBM viability, proliferation and growth, sustaining GBM mitochondrial fitness. Irradiated microglia‐mediated effects on GBM intracellular context result in a stimulation of oxidative phosphorylation mechanism and an increased protection from mitochondrial oxidative stress. Metformin administration inhibits the pro‐tumoural effects mediated by microglial cells on GBM, blocking oxidative phosphorylation metabolic process.

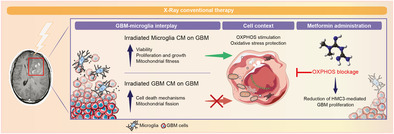

## 1 INTRODUCTION

Glioblastoma (GBM) is the most aggressive primary malignant brain tumour affecting the adult population, with a prognosis that remains dramatically poor and about the 5% of patients survive 5 years after diagnosis.^1^ GBM heterogeneity limits the efficacy of the current therapeutic approaches including surgical resection, followed by radiotherapy (RT) and temozolomide chemotherapy.^2^ Therefore, developing new therapeutic strategies and revealing the mechanisms responsible for the failure of current therapies, become undisputable to improve the outcomes of this devastating disease.

The effects induced by RT on GBM tumour microenvironment (TME) represent a critical field of investigation and the main factor in inducing therapeutic failure.^3^, ^4^ TME of GBM is an intricate network where, in a hypoxic milieu, different cell types coexist, including tumour cells, immune cells, fibroblasts, resident glial and endothelial cells, releasing various secretory factors.^5^, ^6^ RT triggers specific responses within GBM TME, such as cell death, senescence, activation, survival and migration.^7^, ^8^

Tumour‐associated microglia and macrophages (TAMs) are the most abundant non‐neoplastic cells in the TME of GBM. They consist of both brain‐resident microglia and bone marrow‐derived myeloid cells from the periphery, constituting about 40% of the tumour mass in GBM.^9^ In particular, the feedback from microglia, activated by related inflammatory signalling is imprinted by the TME, playing a central role in favouring immunosuppression and immune escape mechanisms promoting tumour resistance.^8^, ^10^

It has been reported that metformin acts as anti‐cancer agent within the complex microenvironment of cancer, particularly in breast cancer.^11^ Metformin belongs to biguanide pharmacological class and represents a first‐line therapy for Type 2 diabetes.^12^ This drug acts reducing gluconeogenesis process and stimulating glucose uptake and consumption.^13^ Regarding tumour suppressor mechanisms, metformin is involved in altering cell metabolism, blocking mitochondrial respiratory chain complex I and inhibiting the tricarboxylic acid cycle and oxidative phosphorylation.^14^ Moreover, metformin sensitizes cells to temozolomide, inhibits cell proliferation and invasion, and decreases hypoxia‐inducible factor and vascular endothelial growth factor, key elements for GBM angiogenesis and malignance.^15^, ^16^

Here, we investigated the irradiation‐induced alterations on microglia and the indirect effects mediated by off‐target irradiation on spared GBM cells, aiming at highlighting exploitable mechanisms to improve tumour control and increasing radiosensitivity.

## 2 MATERIALS AND METHODS

### 2.1 Cell cultures, conditioned media and treatments

Experiments were performed using U‐87 MG and U‐251 MG human GBM cell lines and HMC3 human microglia cell line. GBM cells were purchased from the European Collection of Authenticated Cell Cultures (ECACC, Public Health England, Porton Down Salisbury, UK) and cultured with growth medium [Dulbecco's Modified Eagle Medium (DMEM) high glucose supplemented with 10% foetal bovine serum (FBS), Penicillin–Streptomycin 100 IU/mL and L‐glutamine 2 mmol/L]. HMC3, were purchased from European Collection of Authenticated Cell Cultures (ECACC, Public Health England, Porton Down Salisbury, UK) and cultured with HMC3 growth medium [Minimum Essential Medium (MEM) supplemented with 10% FBS, Penicillin–Streptomycin 100 IU/mL and L‐glutamine 2 mmol/L]. Cells were maintained in growing culture condition in an incubator at 37°C in a humidified atmosphere (95% air and 5% CO_2_). Conditioned media (CM) were collected from U‐87 MG, U‐251 MG, and HMC3 cultures at 24 or 48 h post‐irradiation with 0 Gy (mock‐IR), 2 and 15 Gy doses of X‐ray irradiation, filtered with a 0.22 μm syringe filter unit and stored at – 80°C until use. For boiled CM, CM were incubated at 100°C for 10 min. Then, CM and/or boiled CM were used at a final concentration of 25% with growth medium to culture U‐87 MG or U‐251 MG cell lines in experimental settings according to the described procedures. Metformin (1,1‐dimethylbiguanide hydrochloride, Cat no. 317240, Sigma‐Aldrich) was prepared as a stock solution at 40 mM and stored at −20°C until use. For cell treatment, metformin was diluted at a final concentration of 100 μM in phosphate buffered saline (PBS). The effects of metformin were tested in GBM cell lines cultured with growth medium, irradiated GBM cells CM, or irradiated HMC3 CM. For clonogenic assay, vehicle (i.e., PBS) or metformin were administrated every 48 h. All experiments employed cells at a passage *n* < 25.

### 2.2 RNA extraction and RT‐qPCR for gene expression analysis

Total RNA was extracted by Trizol® reagent following manufacturer's instructions (Invitrogen). First‐strand cDNA was then synthesized with reverse transcription kit (Applied Biosystem). Quantitative real‐time PCR was performed in Step One Fast Real‐Time PCR System, using the SYBR Green PCR MasterMix (Life Technologies). The specific PCR products were detected by SYBR Green fluorescence. The relative mRNA expression level was calculated by the threshold cycle (Ct) value of each PCR product and normalized with that of ACTB by using a comparative 2^−ΔΔCt^ method.^17^ The sequence of primers used is given in Table S1.

### 2.3 Immunocytochemistry analysis

Immunocytochemistry was carried out as previously reported.^18^, ^19^ Briefly, cells were irradiated with 0 Gy (i.e., mock‐IR control group) and 15 Gy doses of irradiation, or exposed to irradiated HMC3 CM for 24 h. U‐87 MG and U‐251 MG cell lines were stained with 200 nM MitoTracker Red CMXRos probe (Thermo Fisher Scientific) for 30 min at 37°C in order to detect mitochondria, according to the manufacturer's instructions. The dye was removed and cells were washed three times in PBS. Nuclei were stained with NucBlue 2% in PBS (Thermo Fisher Scientific, Milan, Italy) for 15 min at 37°C. High‐content analysis on cell cultures was performed using Operetta (Perkinelmer). Images were acquired at 24 h after treatment and quantifications of MitoTracker mean fluorescence intensity (MFI), mitochondrial fragmentation, and mitochondrial integrity were obtained using Operetta Harmony software (Perkinelmer).

For immunocytochemistry analysis on U‐251 MG cell line, cells were seeded in cover glass placed into multi‐well 24 plates at final density of 2 × 10^4^ cells/cm^2^. Cells were fixed in 4% paraformaldehyde at room temperature for 10 min. Then, cells were incubated with blocking solution (10% normal goat serum [NGS] in PBS) for 1 h at room temperature. Samples were then incubated overnight at 4°C with the following primary antibodies diluted in incubating solution (1% NGS in PBS): rabbit anti‐KI‐67 (1:200, Cat no. ab15580, RRID: AB_443209, Abcam, Cambridge, UK), chicken anti‐NESTIN (1:500; Cat no. ab134017, RRID: AB_2753197, Abcam, Cambridge, UK). Then, after 3 washes in PBS, samples were incubated for 1 h at room temperature with an appropriate combination fluorescence of goat secondary antibodies: Goat anti‐rabbit, Alexa Fluor 546 (1:1000, Cat no. A11010, RRID: AB_143156, Invitrogen, Waltham, MA, USA), Goat anti‐chicken, Alexa Fluor 488 (1:1000, Cat no. Ab150169, RRID: AB_2636803, Abcam, Cambridge, UK). Nuclei were counterstained with 4′,6‐diamidino‐2‐phenylindole (1:1000, Cat no. D1306, Invitrogen) for 5 min at room temperature. Slides were mounted with fluorescent mounting medium Permafluor (ThermoScientific) and digital images were acquired using Leica TCS SP8 confocal microscope.

### 2.4 Lactate dehydrogenase assay

The relative cytotoxicity was assessed using lactate dehydrogenase (LDH) activity assay (Abcam), following the manufacturer's instructions. Briefly, cells were seeded in 96‐well plates (Costar) at a final density of 1 × 10^4^ cells/well/100 μL. In order to assess cytotoxicity induced by CM, we performed LDH assay at 24 h post‐CM treatment. Cells were treated with either homocellular or heterocellular CM collected at 24 or 48 h post‐irradiation. Cells treated with 1% of lysis solution (10% triton X‐100 in PBS) were used as positive controls (100% relative cytotoxicity). Vehicle‐treated cells were used as negative control (0% relative cytotoxicity). At given timepoints, quantification of the LDH activity was performed on supernatants following manufacturer's instructions. The absorbance was measured using a Multiskan SkyHigh Microplate spectrophotometer (Thermo Scientific, Milan, Italy) at 450 nm. The percentage of relative cytotoxicity was calculated using the following formula:

$$\%\text{relative cytotoxicity}=\left[\frac{\left(\text{ODsample}–\text{ODnegative control}\right)}{\left(\text{ODpositive control}–\text{ODnegative control}\right)}\right]\times 100.$$

### 2.5 3‐(4,5‐Dimethylthiazol‐2‐yl)‐2,5‐diphenyltetrazolium bromide turnover

For 3‐(4,5‐dimethylthiazol‐2‐yl)‐2,5‐diphenyltetrazolium bromide (MTT) turnover, MTT at a final concentration of 1 mg/mL was added to each well and incubated at 37°C in a humidified atmosphere (5% CO_2_) for 2 h and 30 min under standard culture conditions, as previously described.^20^ MTT turnover was evaluated at 24 and 72 h post‐CM treatment. Then, media were gently removed, 200 μL of MTT solvent (dimethyl sulfoxide [DMSO], Sigma) were added and placed on an orbital shaker for 10 min at room temperature. The absorbance was measured using a Multiskan SkyHigh Microplate spectrophotometer (Thermo Scientific, Milan, Italy) at 570 nm. Metabolic turnover was calculated as:

$$\%\mathrm{MTT}\;\text{turnover}=\left(\frac{\mathrm{OD}\;\text{sample}}{\text{average control}}\right)\times 100.$$

Cells cultured with 0 Gy CM, derived from both GBM and HMC3 cell cultures, were used as positive control (100% MTT turnover). Each experiment was performed three times, with *n* > 4 replicates per condition during each experimental run.

### 2.6 Clonogenic assay

Clonogenic assay was performed on U‐87 MG or U‐251 MG cell lines. 400 cells (U‐87 MG) or 600 cells (U‐251 MG) were plated in a 6 multi‐well plates with a culture surface of 9.5 cm^2^ per well. Cells were incubated with 2 mL of either 100% growth medium or 25% CM and 75% growth medium. For both naïve U‐87 MG and naïve U‐251 MG, the following experimental conditions were tested: 0 Gy irradiated GBM and HMC3 CM, 15 Gy GBM and HMC3 irradiated CM, 0 Gy irradiated HMC3 CM boiled, 15 Gy irradiated HMC3 CM boiled. Clonogenic growth was allowed for 13 days for U‐87 MG and 7 days for U‐251 MG. Cells were then fixed with methanol for 15 min at room temperature. Colonies were stained with 1% crystal violet for 25 min at room temperature.^21^ Colonies which accounted for more than 50 cells were considered as clones. Plating efficiency (PE) of controls was calculated as:

$$\text{PEcontrol}=\frac{\text{number of clones}}{\text{number cell plated}}$$

The percentage of surviving fraction was calculated as:

$$\%\text{of surviving fraction}=\left(\frac{\mathrm{PE}\;\text{sample}}{\mathrm{PE}\;\text{control}}\right)\times 100$$

### 2.7 Flow cytometry

For flow cytometry‐assisted viability analysis, 2.5 × 10^5^ cells were plated in T25 cell plate with a culture surface of 25 cm^2^. GBM and microglia cells were cultured for 24 h and then irradiated with 0, 2 or 15 Gy. After 24 h from radiation treatment, cells were stained in order to asses Annexin V/propidium iodide (PI) assay. GBM cell lines were also treated for 24 h with 0, 2 and 15 Gy irradiated cells‐derived CM. CM were collected after 24 h from x‐ray treatment. After treatments, cells were washed and re‐suspended in 100 μL of PBS at 4°C. 1 μL of Annexin V‐FITC solution and 5 μL of PI (Beckmam Coulter) were added to cell suspension and mixed gently. Cells were incubated for 15 min at room temperature. Finally, 400 μL of binding buffer were added and cell preparation was analysed by flow cytometry (MACSQuant Analyzer 10, Miltenyi Biotech) and analysed using Flowlogic software (Miltenyi Biotech).^22^ To determine the mitochondrial reactive oxygen species (ROS) levels, cells were stained with 2.5 μM of MitoSOX probe for 30 min at 37°C and fluorescence intensity was measured according to the fluorescence detection conditions of PE‐MitoSOX‐A B2‐A using the MACSQuant Analyser (Miltenyi Biotech).

### 2.8 Irradiation

Full experimental procedures are described in the Supplementary Data S1. Irradiation was performed in a linear accelerator, Elekta Synergy, at the Radiotherapy Department of Cannizzaro Hospital, Catania, Italy with a dose rate of 3 Gy/min, using a 6 MV x‐ray. GBM cell irradiation was carried out using dose values of 0 Gy (mock‐IR group), 2and 15 Gy.

### 2.9 Statistical considerations

Data were tested for normality using a D'Agostino and Pearson omnibus normality test and subsequently assessed for homogeneity of variance. Data that passed both tests were further analysed by two‐tailed unpaired Student's *t*‐test, that was used for comparison of *n* = 2 groups. For comparison of *n* ≥ 3 groups, one‐way analysis of variance, followed by Holm–Sidak post hoc test for multiple comparisons were used. All tests were performed using GraphPad Prism (version 5.00 for Mac, GraphPad Software). For all statistical tests, *p*‐value <0.05 was considered statistically significant and symbols indicating statistical differences are reported in figure legends.

## 3 RESULTS

### 3.1 Irradiated HMC3 CM preserve GBM cells viability

**FIGURE 1:**
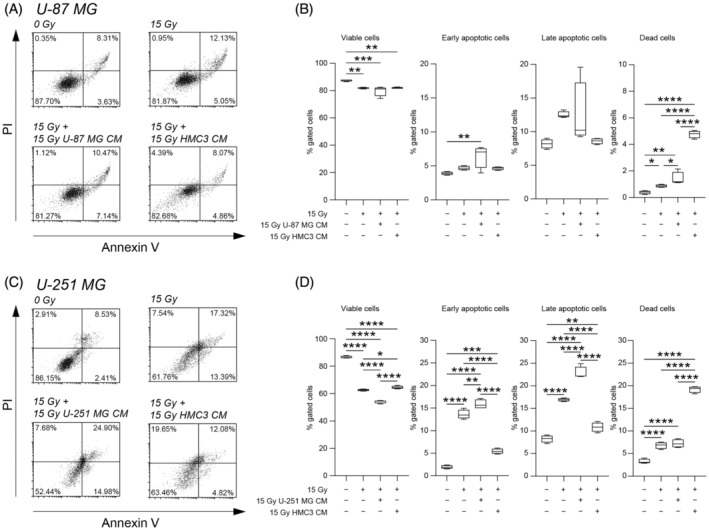
Direct irradiation and homocellular or heterocellular conditioned media (CM)‐derived from irradiated cells reduce glioblastoma cell lines viability. (A,B) Cytofluorimetric analysis of viability evaluated with Annexin V/propidium iodide (PI) assay on U‐87 MG cell line. (C,D) Cytofluorimetric analysis of viability evaluated with Annexin V/PI assay on U‐251 MG cell line. Data are shown via standard box and whiskers and viability is expressed as the percentage of gated cells, *n* = 4 independent replicates for each experimental condition. *p‐value <0.05; **p‐value <0.01; ***p‐value <0.001; ****p‐value <0.0001.

We first evaluated the effects of irradiation on U‐87 MG and U‐251 MG cell viability at 24 h post‐treatment, using a cytofluorimetric assisted Annexin V/PI assay (Figure 1A‐D). Mock‐IR cells (i.e., 0 Gy) were used as controls. As expected, our results suggested that 15 Gy dose induced a significant reduction of the percentage of viable cells in all tested cell lines as compared to mock‐IR cells (Figure 1). Similar effects were observed when human microglial cell line was exposed to direct irradiation (Figure S1). We did not observe any significant changes in the proportion of viable cells in U‐87 MG exposed to 15 Gy IR with CM derived from 15 Gy U‐87 MG or from 15 Gy HMC3 cells as compared to 15 Gy U‐87 MG (Figure 1A,B). Similar results were confirmed on U‐251 MG cell line, in which CM derived from 15 Gy U‐251 MG cells induced a significant reduction in the proportion of viable cells as compared to mock‐IR, 15 Gy irradiated and 15 Gy irradiated treated with CM derived from 15 Gy HMC3 (Figure 1C,D). In an effort to find potential effects of direct irradiated and irradiated‐derived CM treatment, we performed an exploratory KI‐67 immunocytochemistry analysis on U‐251 MG cells at 0 Gy, 15 Gy, 15 Gy plus 15 Gy U‐251 MG CM and 15 Gy plus 15 Gy HMC3 CM (Figure S2). We found a strong reduction of the proportion of KI‐67 positive cells in all directly irradiated conditions, independently from CM exposure, even if a small proportion of cells was found to be KI‐67 positive in HMC3 CM exposed cultures (Figure S2).

**FIGURE 2:**
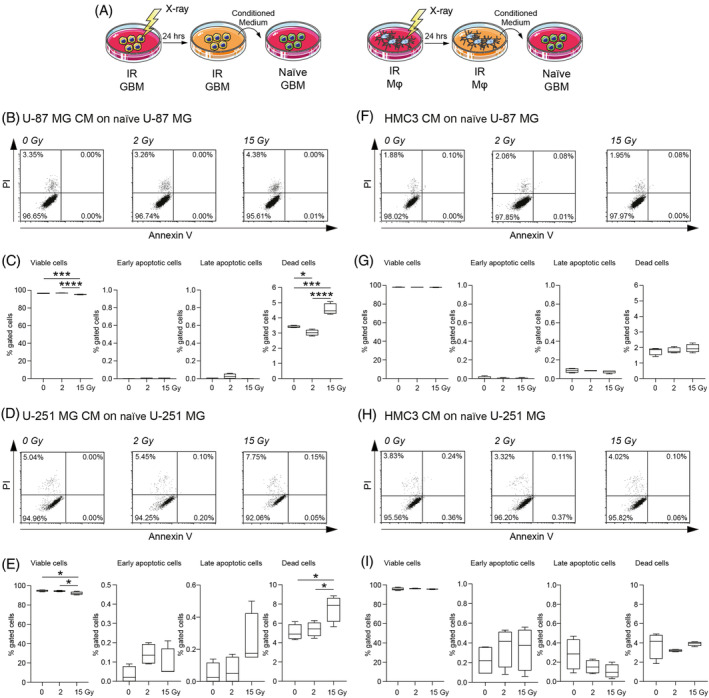
Irradiated HMC3 conditioned media (CM) preserve radiation‐naïve U‐87 MG and U‐251 MG cell lines viability. (A) Experimental workflow and CM treatment. (B–I) Cytofluorimetric analysis of viability evaluated with Annexin V/propidium iodide (PI) assay on U‐87 MG treated with U‐87 MG CM (B,C), U‐251 MG treated with U‐251 MG CM (D,E), U‐87 MG treated with HMC3 CM (F,G) and U‐251 MG treated with HMC3 CM (H,I). Data are shown as standard box and whiskers and viability is expressed as the percentage of gated cells, *n* = 4 independent replicates for each experimental condition. *p‐value <0.05; ***p‐value <0.001; ****p‐value <0.0001. GBM, glioblastoma; Mφ, microglia; IR, irradiated.

In order to evaluate the potential effects of secretome derived from irradiated cells on radiation‐naïve cells, we exposed cell cultures to either 0 Gy (mock‐IR), 2 and 15 Gy irradiation and we collected their CM at 24 h, exposing naïve cells to their media and evaluating cell viability after 24 h of conditioning (Figure 2A). Analysis on U‐87 MG showed an increased proportion of dead cells when treated with 15 Gy U‐87 MG CM as compared to naïve U‐87 MG treated with mock‐IR U‐87 MG CM (Figure 2B,C) and versus 2 Gy U‐87 MG CM‐treated cells (Figure 2B,C). Naïve cells incubated with 2 Gy U‐87 MG CM showed a near‐normal levels of viable, early/late apoptotic, and dead cells as compared to control (Figure 2B,C). We then moved to analyse the effects of CM from irradiated HMC3 on radiation‐naïve U‐87 MG cells. Interestingly, in this case, we did not observe any significant differences in terms of cell viability among groups (Figure 2F,G), indicating that CM of irradiated microglia do not influence U‐87 MG cells viability. We also assessed this effect on U‐251 MG cells (Figure 2D,E,H,I). We found that 15 Gy U‐251 MG CM induced a decrease of cell viability as compared to the mock‐IR U‐251 MG CM (Figure 2D,E) and also versus 2 Gy U‐251 MG CM (Figure 2D,E), coupled with a significantly increased proportion of dead cells in 15 Gy U‐251 MG CM treated cells as compared to 2 Gy and mock‐IR U‐251 MG CM of about 1.6 fold (Figure 2D,E). It may be possible that the differences in culture media may slightly affect control cultures viability, even if more than 95% of cells were viable in mock‐IR cultures.

Finally, we assessed the effects on cytotoxicity and metabolic turnover after incubation with irradiated GBM and microglia CM on U‐87 MG and U‐251 MG cell lines, using LDH and MTT assays at different timepoints (Figure S3). LDH assay revealed limited cytotoxicity in cells cultured with irradiated CM and similar results were observed for MTT turnover at 24 h (Figure S3). Notably, CM collected 48 hours post‐irradiation, induced an increased relative cytotoxicity in U‐87 MG CM treated naïve U‐87 MG and U‐251 MG CM treated naïve U‐251 MG, but not in cells treated with HMC3‐derived CM (Figure S4).

### 3.2 15 Gy irradiated HMC3 CM stimulate GBM clone formation

**FIGURE 3:**
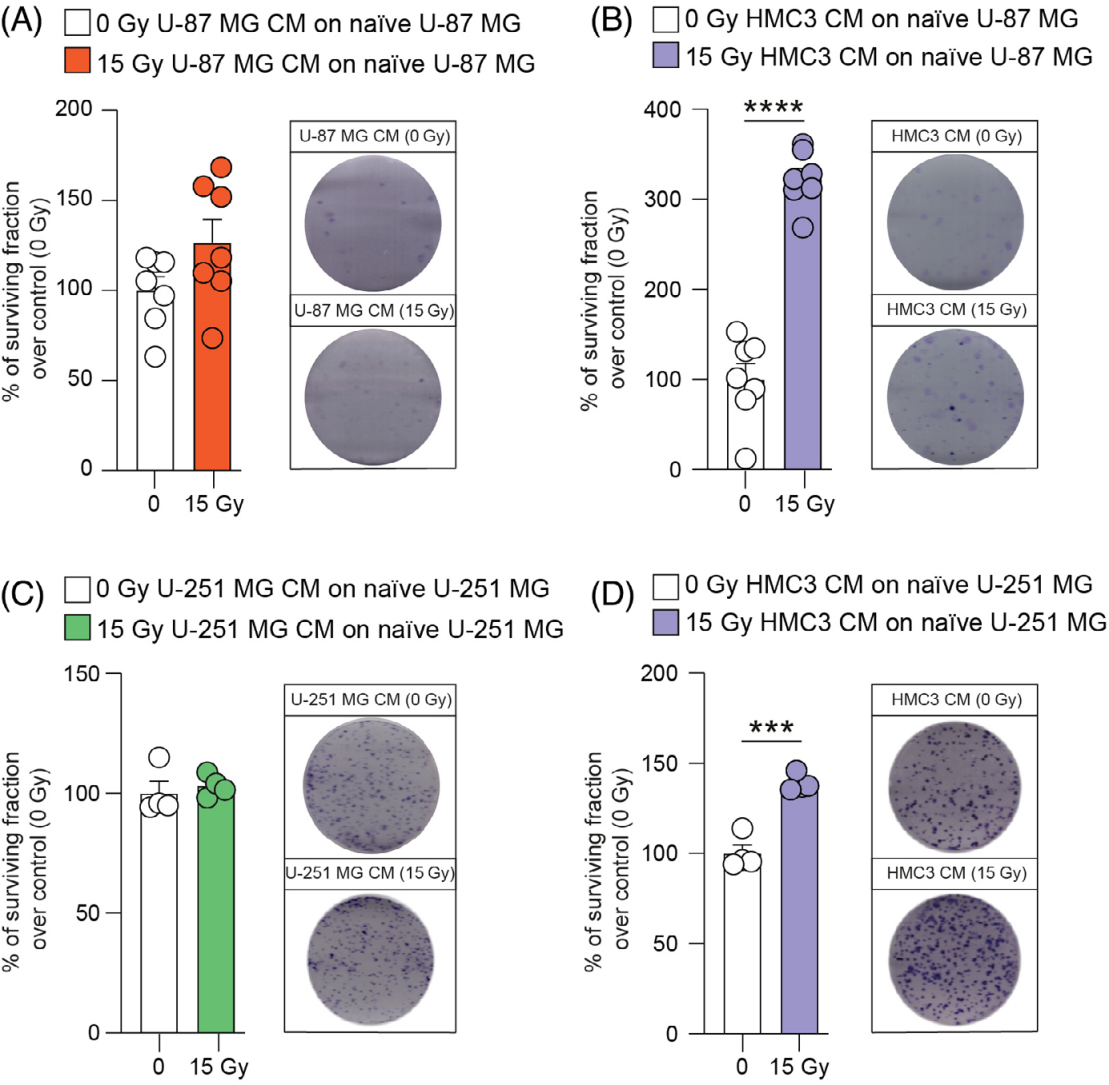
Irradiated HMC3 conditioned media (CM) stimulate naïve U‐87 MG and U‐251 MG clone formation. (A,B) Surviving fraction and representative pictures of U‐87 MG treated with 0 Gy and 15 Gy U‐87 MG CM (A) or 0 Gy and 15 Gy HMC3 CM (B). (C,D) Surviving fraction and representative pictures of U‐251 MG treated with 0 Gy and 15 Gy U‐251 MG CM (C) or 0 Gy and 15 Gy HMC3 CM (D). Data are expressed as scattered dot‐plot and mean ± SEM of *n* ≥ 3 independent experiments. ***p‐value <0.001 and ****p‐value <0.0001.

In order to find a potential effect on clonogenicity of GBM cells induced by homocellular or heterocellular communication via CM, we performed a clonogenic assay on U‐87 MG and U‐251 MG cells. Interestingly, 15 Gy U‐87 MG CM were not able to induce significant effects on radiation‐naïve U‐87 MG cells (Figure 3A). Conversely, an increase of the surviving fraction was observed in radiation‐naïve U‐87 MG cells treated with 15 Gy HMC3 CM as compared to mock‐IR HMC3 CM (Figure 3B). Similar results were observed on U‐251 MG cell line, in which 15 Gy U‐251 MG CM induced clone formation comparable to control cultures (Figure 3C). A significant increase on U‐251 MG surviving fraction was detected after treatment with 15 Gy HMC3 CM versus control treated with mock‐IR HMC3 CM (Figure 3D). In addition, analysis of fresh versus boiled CM derived from HMC3 showed no significant differences (Figure S5), indicating that the effects observed are mediated by thermo‐stable molecules.

### 3.3 Irradiated HMC3 CM sustain mitochondrial fitness in GBM

In order to analyse mitochondrial fitness, we evaluated the effects of direct irradiation on GBM cell lines treated with mock‐IR or 15 Gy HMC3 CM. We used a high‐content analysis of mitochondrial mass, mitochondrial fragmentation and mitochondrial integrity on mock‐IR versus 15 Gy directly irradiated GBM cells. Analysis on whole cell mitochondrial mass showed no significant differences between tested conditions on both U‐87 MG and U‐251 MG, although a slight decrease of mitochondrial fragmentation was observed in 15 Gy U‐251 MG (Figure S6). Interestingly, HMC3 that underwent direct irradiation, showed a significant decrease of both mitochondrial mass (Figure S6) and mitochondrial fragmentation as compared to mock‐IR HMC3 (Figure S6).

**FIGURE 4:**
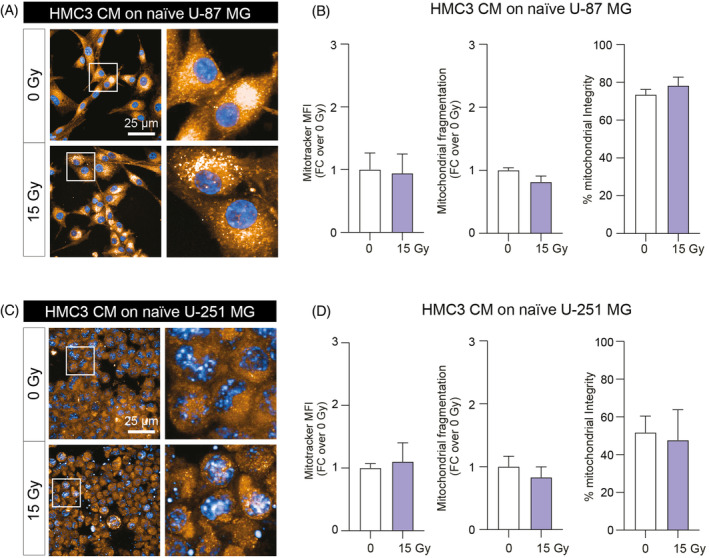
Irradiated HMC3 conditioned media (CM) do not influence mitochondrial mass, fragmentation and integrity in naïve U‐87 MG and U‐251 MG cells. (A,B) Representative pictures of immunofluorescence staining with Mitotracker of U‐87 MG treated with 0 or 15 Gy HMC3 CM (A) and high‐content analysis of Mitotracker mean fluorescence intensity (MFI), mitochondrial fragmentation and percentage of mitochondrial integrity (B). (C,D) Representative pictures of immunofluorescence staining with Mitotracker of U‐251 MG treated with 0 or 15 Gy HMC3 CM (C) and high‐content analysis of Mitotracker MFI, mitochondrial fragmentation and percentage of mitochondrial integrity (D). Data are shown as bar plot and expressed as mean ± SEM of *n* = 4 independent replicates.

In order to analyse the effect of irradiated HMC3 CM treatment on mitochondrial fitness of GBM cell lines, we tested the CM‐induced effects on U‐87 MG and U‐251 MG mitochondrial state and structure after 24 h incubation with either mock‐IR and 15 Gy HMC3 CM (Figure 4). Our results suggested that HMC3 CM treatment had no significant effects of mitochondrial mass, fragmentation and on percentage of intact mitochondria in tested GBM cell lines, U‐87 MG (Figure 4A,B) and U‐251 MG (Figure 4C,D), indicating that HMC3 CM do not affect mitochondrial function, fitness and preserve mitochondrial mass at near‐normal levels.

### 3.4 HMC3 CM protect radiation‐naïve GBM cell lines from mitochondrial oxidative stress

**FIGURE 5:**
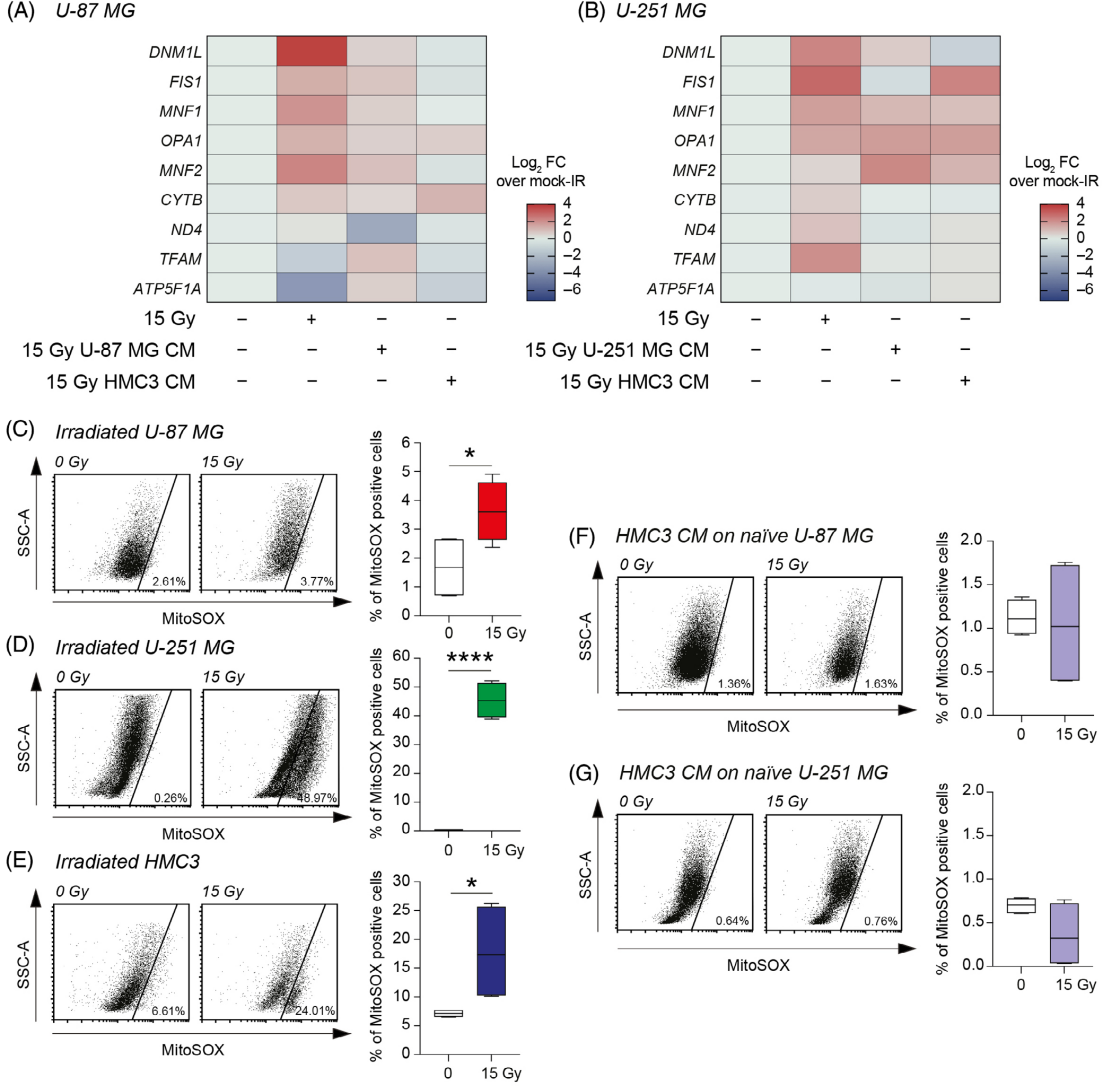
Radiation increases mitochondrial reactive oxygen species and irradiated HMC3 conditioned media (CM) preserve mitochondrial oxidative state of U‐87 MG and U‐251 MG cells. (A) qRT‐PCR analysis of mRNA expression levels of DNM1L, FIS1, MNF1, OPA1, MNF2, CYTB, ND4, TFAM, and ATP5F1A in mock‐IR U‐87 MG, 15 Gy U‐87 MG and mock‐IR treated with 15 Gy U‐87 MG CM or 15 Gy HMC3 CM; data are shown as Log_2_ FC over mock‐IR; (B) qRT‐PCR analysis of mRNA expression levels of DNM1L, FIS1, MNF1, OPA1, MNF2, CYTB, ND4, TFAM and ATP5F1A in mock‐IR U‐251 MG, 15 Gy U‐251 MG and mock‐IR treated with 15 Gy U‐251 MG CM or 15 Gy HMC3 CM; data are shown as Log_2_ FC over mock‐IR; (C–E) Cytofluorimetric analysis of MitoSOX positive cells in 0 or 15 Gy U‐87 MG (C), 0 or 15 Gy U‐251 MG (D) and 0 or 15 Gy HMC3 (E). (F,G) Cytofluorimetric analysis of MitoSOX positive cells in U‐87 MG cultured with 0 or 15 Gy HMC3 CM (F) and in U‐251 MG cultured with 0 or 15 Gy HMC3 CM (G). Data in (C–G) are shown via standard box and whiskers and are expressed as percentage of MitoSOX positive cells of *n* = 4 replicates for each experimental condition. *p‐value <0.05; ****p‐value <0.0001.

In order to clarify the involvement of mitochondria and the effects observed on GBM cells cultured in irradiated microglia CM, we moved to evaluate the mRNA expression levels of the main genes involved in mitochondrial fusion, fission and stability. We analysed the levels of dynamin 1 like (DNM1L), fission, mitochondrial 1 (FIS1), ubiquinol‐cytochrome c reductase complex assembly factor 2 (MNF1), OPA1 mitochondrial dynamin‐like GTPase (OPA1), mitofusin 2 (MNF2), cytochrome b (CYTB), NADH dehydrogenase subunit 4 (ND4), transcription factor A, mitochondria (TFAM), and ATP synthase F1 subunit alpha (ATP5F1A) on mock‐IR U‐87 MG and U‐251 MG, 15 Gy irradiated cells or mock‐IR cells exposed to 15 Gy irradiated homocellular (U‐87 MG or U‐251 MG, respectively) or heterocellular (i.e., HMC3) CM (Figure 5A,B). On the one hand, we observed similar irradiation signature in both U‐87 MG and U‐251 MG exposed to 15 Gy direct irradiation, with increase of DNM1L, FIS1, MNF1, OPA1, and MNF2 as compared to mock‐IR controls (Figure 5A,B). On the other hand, homocellular and heterocellular CM exposure induced less evident effects and mRNA expression levels similar to mock‐IR controls (Figure 5A,B).

To assess the role of mitochondrial ROS, we analysed the MitoSOX levels on directly irradiated cells finding a significant increase of the proportion of MitoSOX positive cells in all tested cell lines irradiated with 15 Gy as compared to mock‐IR (Figure 5C–E). Particularly, we observed an increased MitoSOX positive cells proportion in all tested irradiated cells versus mock‐IR cells (Figure 5C–E). Notably, mitochondrial ROS evaluation on 15 Gy HMC3 CM‐treated GBM cell lines showed no significant changes as compared to mock‐IR HMC3 CM group (Figure 5F,G), indicating that CM from irradiated microglia do not affect ROS production on radiation‐naïve GBM cells.

### 3.5 Metformin administration reduces GBM clone formation mediated by irradiated HMC3 CM

**FIGURE 6:**
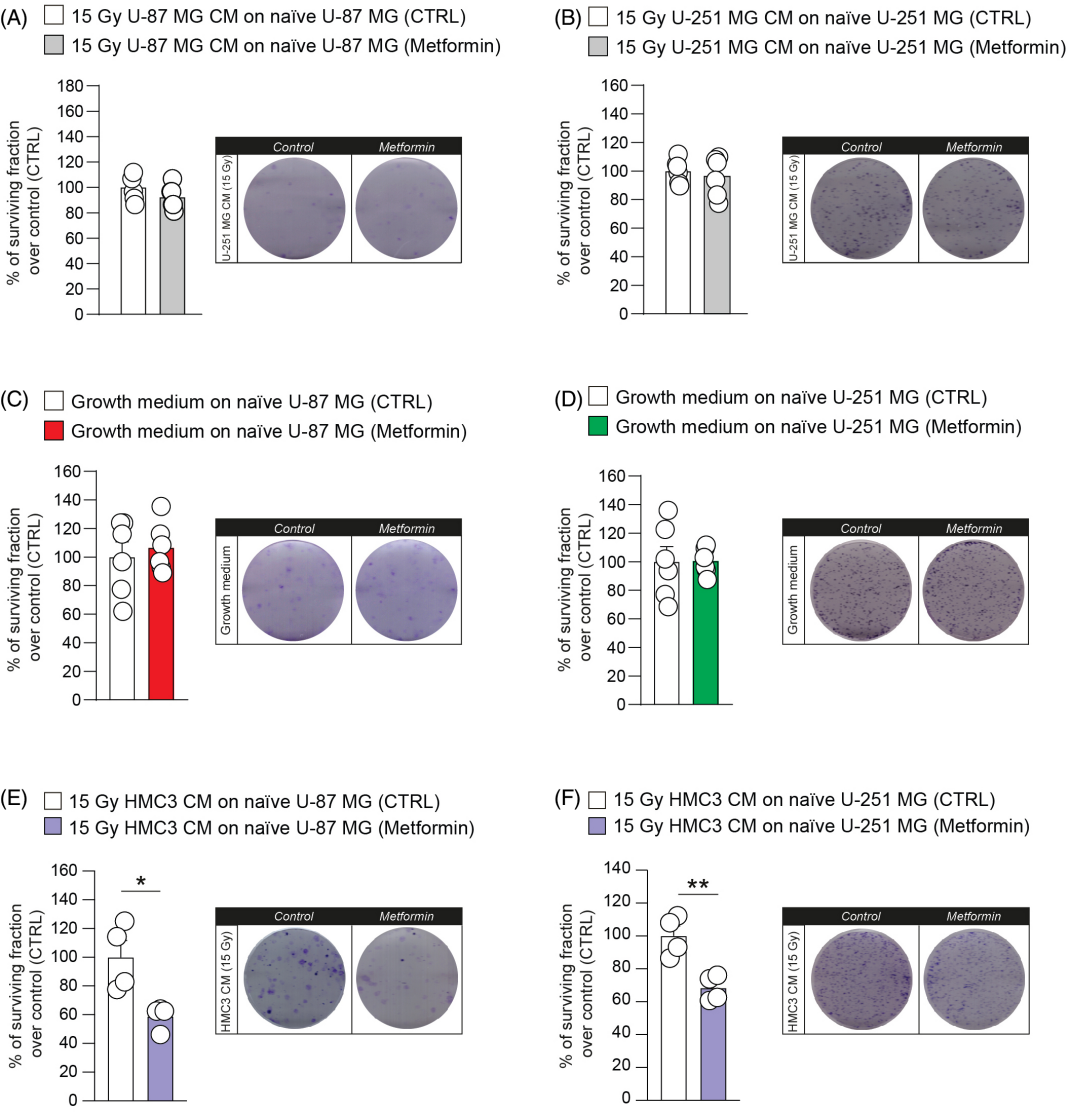
Metformin administration reverses irradiated HMC3 conditioned media (CM)‐induced effects. (A,B) Surviving fraction and representative pictures of U‐87 MG (A) and U‐251 MG (B) cell lines ± metformin, cultured with 15 Gy GBM CM. (C,D) Surviving fraction and representative pictures of U‐87 MG (C) and U‐251 MG (D) cell lines ± metformin, cultured with standard growth medium. (E,F) Surviving fraction and representative pictures of U‐87 MG (E) and U‐251 MG (F) cell lines ± metformin, cultured with 15 Gy HMC3 CM. Data are expressed as scattered dot‐plot and mean ± SEM of *n* ≥ 4 independent experiments. *p‐value <0.05 and **p‐value <0.01.

In an effort to find potential modulators of heterocellular communication mediated by CM of irradiated HMC3, we tested whether oxidative phosphorylation mechanisms were involved in this phenomenon. We performed a clonogenic assay on U‐87 MG and U‐251 MG cell lines cultured with irradiated GBM CM (Figure 6A,B) and cultured in standard growth medium (Figure 6C,D), treated or not with metformin. Our results showed that metformin administration did not affect clone formation when U‐87 MG and U‐251 MG were cultured in growth medium (Figure 6C,D). A similar result was observed for GBM cell lines exposed to irradiated GBM CM (Figure 6A,B).

We then tested the effects of metformin administration on GBM cells treated with 15 Gy HMC3 CM using clone formation assay (Figure 6E,F). U‐87 MG and U‐251 MG showed a similar response to pharmacological blockage of mitochondrial oxidative phosphorylation system mediated by metformin. Particularly, U‐87 MG showed a reduction of surviving fraction after metformin administration in 15 Gy HMC3 CM (Figure 6E). Similar results were observed in U‐251 MG cell line, showing a significant reduction of the surviving fraction after treatment with metformin in 15 Gy HMC3 CM‐treated U‐251 MG cells (Figure 6F). Taken together, these data indicate that the decreasing of clonogenicity of GBM cell lines treated with irradiated HMC3 CM was a specific downstream result of metformin and that irradiated HMC3 CM stimulate oxidative phosphorylation mechanisms that can be targeted by metformin.

## 4 CONCLUSION

TME acquires different phenotypes and is differentially modulated by TAMs, contributing to GBM molecular subtyping.^23^ Typically, TAMs have a pivotal role in leading tumour progression by limiting T cell‐mediated anti‐tumour immune response and stimulating cell proliferation and angiogenesis.^24^ Metabolic changes are coupled with modifications of oxygen and nutrients availability, resulting from the complex relation among the different cellular components that mediate tumour metabolic re‐wiring.^25^ This condition appears radical in GBM, so much so that is possible to distinguish GBM subtypes from a metabolic point of view, identifying mitochondrial GBM subset mainly based on oxidative mechanisms, characterized by an increase of oxidative phosphorylation process at the expense of glycolysis.^26^ Therefore, controlling the metabolism both in cancer and resident central nervous system (CNS) cells is a promising approach to limit resistance to therapy and/or to sensitize tumour.^27^

Herein, we focused on the alteration induced by irradiation on TME, especially examining microglia‐GBM interplay. Our results are in accordance to previously reported evidence on RT‐induced GBM recurrences and proliferation^28^ and expand on the biological effects of irradiated microglia via extracellular milieu‐mediated signalling. We found that microglial cells release factors that protect and preserve GBM viability, limiting apoptotic and cell death processes that affect radiation‐naïve GBM cells. This effect was not observed when radiation‐naïve GBM cells were treated with irradiated GBM CM, thus indicating a specific heterotypic‐heterocellular communication between microglia and GBM. We also confirm that irradiated microglia act as a promoter of GBM proliferation through the secretion of thermo‐stable molecules, ruling out the hypothesis that such an effect is primarily induced by protein mediators release in the surrounding milieu.

The hypothesis of a potential influence of TAMs on GBM metabolism and energy state is currently a largely unexplored field. Recent evidence suggests that metabolic alterations following RT may be linked to potential tumour‐permissive changes and may be related to recurrences.^4^ Given the high impact of metabolism in tumour growth, migration and resistance to therapy, we sought to evaluate the metabolic state of GBM cells.

It is well established that RT promotes mitochondrial rearrangement, as a mechanism related to cell stress response.^29^ Mitochondria‐related morphology, metabolism, respiration and ROS production are largely involved in RT‐mediated alterations.^30^ Therefore, we assume that microglia prevent and protect directly RT‐mediated effects, as bystander mechanisms promoted by RT on tumour mass. This assumption correlates with evidence that RT mediates direct effects on GBM cells, and also off‐target bystander effects, modifying both TME cell composition and tumour.^31^

Furthermore, we demonstrate that HMC3 CM induces GBM proliferation stimulating oxidative phosphorylation mechanisms. This effect was not observed in GBM CM‐induced process. In fact, metformin reduces GBM clone formation in radiation‐naïve tumour cells cultured with irradiated HMC3 CM as compared to controls. As such, we speculate that microglia act as a promoter of GBM proliferation, at least in vitro, through the stimulation of respiration mechanisms, known as ‘the reverse Warburg effect’. This mechanism may represent an adaptive response that modulates tumour cell metabolism according to the composition and state of surrounding cell populations.^25^, ^32^

Our study would benefit from future research on in vivo models of GBM and the effect of RT on either cancer cells or CNS‐resident cell populations. Moreover, a focus on mitochondrial DNA and mitochondria transfer via connexins/gap junction, crucial for cell‐to‐cell interaction,^33^ or by subcellular transporting mechanisms, such as tunnelling nanotubes and microvesicles or extravesicles, would add significant information on the underlying biological processes.^25^ It is well documented that microvesicles are secreted by tumour and microglia cells, and they could mediate GBM‐no‐tumoural communication using TME as a medium. Released exosomes may serve as carriers for cell‐to‐cell communication, which affects brain tumour progression and malignancy and controls microglia activation and GBM cell development in an autocrine and paracrine fashion.^34^ Furthermore, in recent years the involvement of immunometabolism has been acquiring an increasing scientific interest in re‐orchestrating GBM TME. Recent studies show how GBM and immune system metabolism interplay may interact to drive immunosuppressive processes.^35^, ^36^ Among the limitations of our study, the identification of specific players in modulating GBM proliferation and metabolism is still elusive, even if we were able to rule out protein mediators and suggest a proof‐of‐principle role of metformin in reducing clonogenicity mediated by irradiated microglia‐derived CM.

We report evidence that could have important consequences for radioresistance mechanisms of GBM and for the molecular processes that increase the post‐RT recurrence rate. There are many in vitro and pre‐clinical studies evaluating the combinatorial approach of temozolomide and disulfiram, which can cross the blood–brain barrier (BBB) and metformin, which has been shown to inhibit GBM stem cells (GSCs) proliferation.^37^ Metformin‐mediated effects as anti‐tumoural agent have been reported for a number of human tumours, including GBM.^38^ Indeed, it was demonstrated that anti‐cancer metformin‐related effects are not only indirect, related to antagonist role on pro‐tumuoral effects induced by hyperglycaemia, but also directly related to a decrease of tumour growth.^39^ Pharmacological efficacy of metformin was confirmed in terms of reduction of proliferation, survival, clonogenicity, and in vivo tumorigenicity of GSCs. In conclusion, metformin, due to the ability to cross BBB, reveals a valuable and promising therapeutic tool for GBM treatment.^40^

## AUTHOR CONTRIBUTIONS

*Conceptualization*: C.A., F.T., S.D.A., D.T., N.V., and R.P. *Methodology*: C.A., F.T., S.D.A., L.L., S.G., S.M., M.G.S., F.P.C., G.R., A.S.A., G.L.V., D.T., N.V., and R.P. *Investigation*: C.A., F.T., S.D.A., L.L., S.G., G.S., G.M., S.M., F.P.C., G.R., A.S.A., A.Z., D.L.F., A.S.A., G.L.V., D.T., and N.V. *Formal analysis*: C.A., F.T., S.D.A., L.L., S.G., R.G., G.L.V., D.T., N.V., and R.P. *Supervision*: R.G., G.L.V., D.T., N.V., and R.P. *Writing—original draft*: C.A., N.V., and R.P. *Writing—reviewing and editing and final approval*: all authors.

## FUNDING INFORMATION

C.A. was supported by the international PhD Program in Biotechnology (Department of Biomedical and Biotechnological Sciences, University of Catania, Italy). F.T. was supported by the Fondazione Umberto Veronesi. N.V. was supported by the PON AIM R&I 2014‐2020‐E66C18001240007. A.S.A. was partially supported by the researchers project number (RSPD2024R750) King Saud University, Riyadh, Saudy Arabia. This study was supported by Piano di Incentivi per la Ricerca di Ateneo 2020–2022, Linea di Intervento 2, “MD‐RESETT‐GLIO” to R.P. This study was partially funded by the National Plan for NRRP Complementary Investments (PNC, established with the decree‐law 6 May 2021, n. 59, converted by law n. 101 of 2021) in the call for the funding of research initiatives for technologies and innovative trajectories in the health and care sectors (Directorial Decree n. 931 of 06‐06‐2022) ‐ project n. PNC0000003 ‐ AdvaNced Technologies for Human‐centrEd Medicine (project acronym: ANTHEM). This work reflects only the authors' views and opinions, neither the Ministry for University and Research nor the European Commission can be considered responsible for them.

## CONFLICT OF INTEREST STATEMENT

The authors declare no conflict of interest.

## Supporting information


**Data S1:** Supporting information.

## Data Availability

The datasets used and/or analysed in this study are reported within the article and/or additional files are available from the corresponding authors.
