# Supplementary material for: Microglia and glioblastoma heterocellular interplay sustains tumour growth and proliferation as an off‐target effect of radiotherapy
Source: Cell Prolif. 2024 Mar 7;57(6):e13606. doi: 10.1111/cpr.13606 (PMC11150140; doi:10.1111/cpr.13606)
Supplement: Supplementary file 1 — Data S1: Supporting information. [file CPR-57-e13606-s001.docx]

Supplementary Data

**Cellular interplay in irradiated GBM**


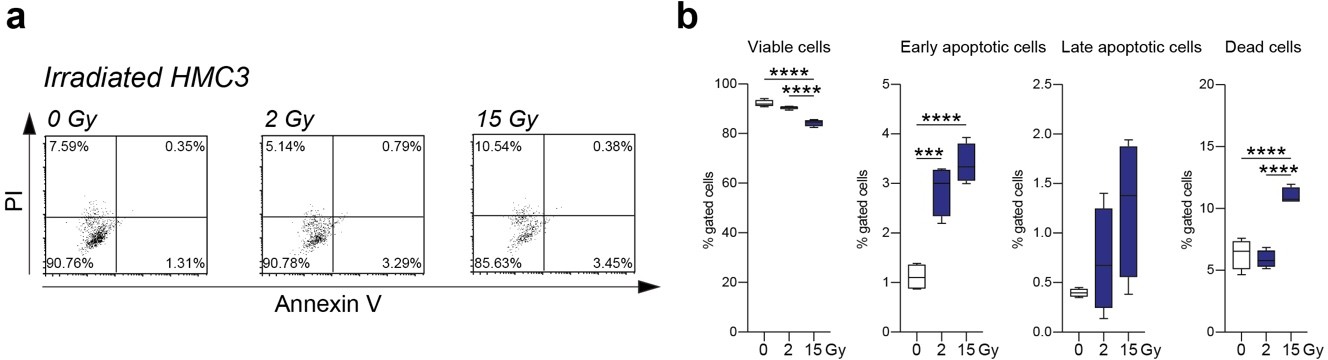


# Supplementary Figure 1. Direct radiation induces apoptosis and cell death in HMC3 cell line. a-

b) Cytofluorimetric analysis of viability evaluated with Annexin V/PI assay on HMC3 cell line. Data are shown via standard box and whiskers and viability is expressed as the percentage of gated cells, n

= 4 independent replicates for each experimental condition. *** p-value < 0.001; **** p-value < 0.0001.


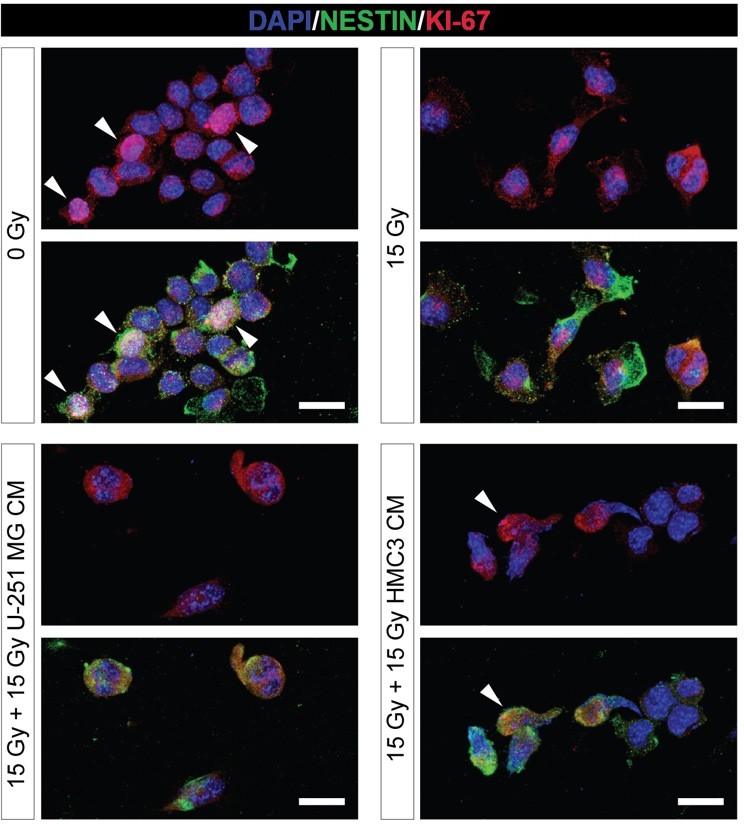


**Supplementary Figure 2. Direct radiation decreases the proportion of KI-67 positive cells.** Representative microphotographs of NESTIN (as a population marker, green cytoplasmatic signal) and KI-67 (as a proliferation marker, red nuclear signal) immunocytochemical analysis of mock-IR U-251 MG cells, 15 Gy U-251 MG, 15 Gy U-251 MG cells treated with 15 Gy U-251 MG CM and 15 Gy U- 251 MG treated with 15 Gy HMC3 CM; nuclei were counterstained with DAPI; scale bar = 10 µm.


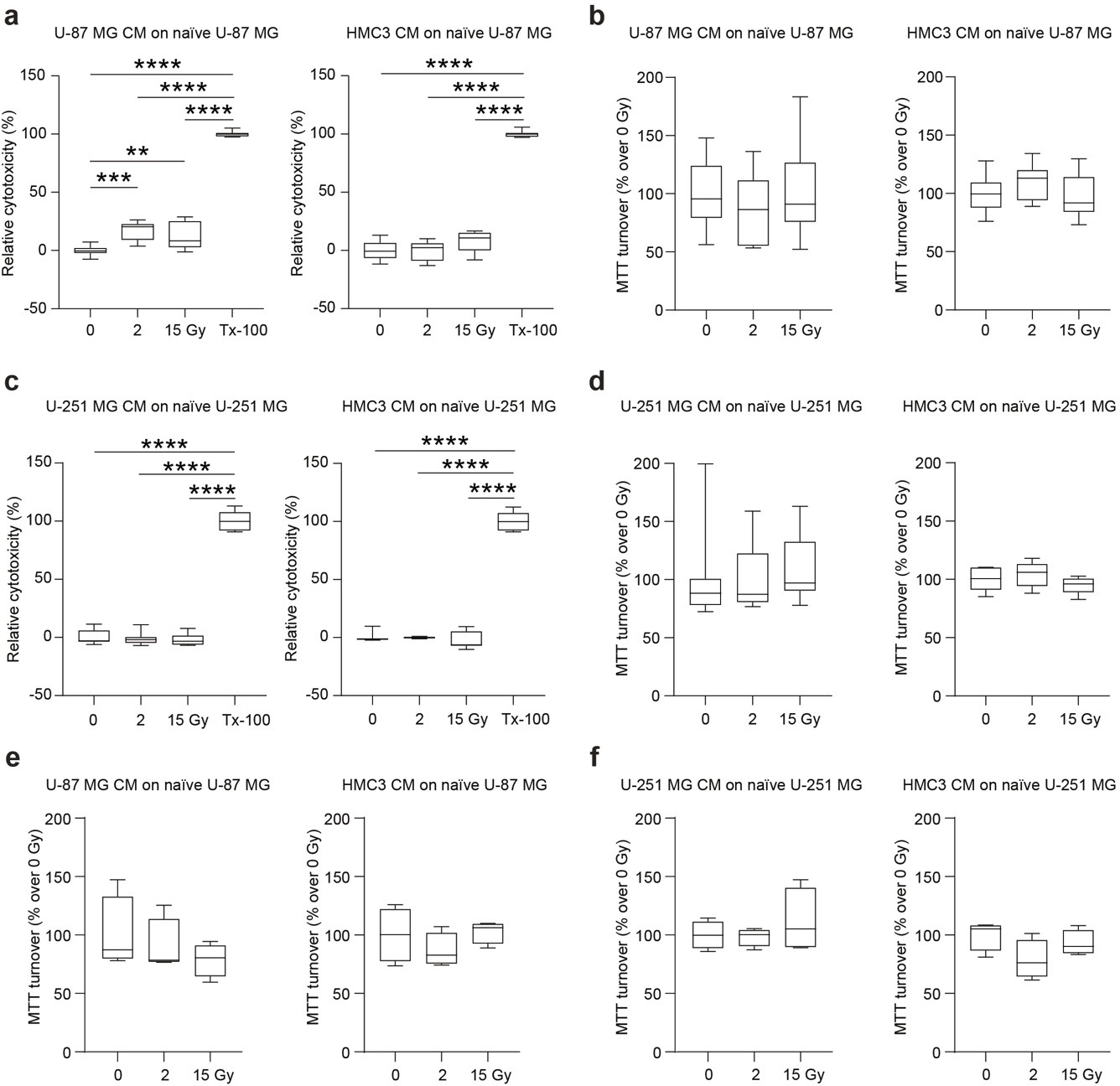


**Supplementary Figure 3. Irradiated HMC3 CM treatment on U-87 MG and U-251 MG cell lines maintains cell viability and preserves metabolic turnover**. a) LDH viability assay on U-87 MG cultures treated with 0 Gy, 2 Gy or 15 Gy U-87 MG CM or HMC3 CM. b) MTT turnover of U-87 MG treated with 0 Gy, 2 Gy or 15 Gy U-87 MG CM or HMC3 CM. c) LDH viability assay on U-251 MG cultures treated with 0 Gy, 2 Gy or 15 Gy U-251 MG CM or HMC3 CM. d) MTT turnover of U-251 MG treated with 0 Gy, 2 Gy or 15 Gy U-251 MG CM or HMC3 CM. e) MTT turnover of U-87 MG treated with 0 Gy, 2 Gy or 15 Gy U-87 MG CM or HMC3 CM for 72 hours. f) MTT turnover of U- 251 MG treated with 0 Gy, 2 Gy or 15 Gy U-251 MG CM or HMC3 CM for 72 hours. Data are shown via standard box and whiskers of n ≥ 4 independent replicates for each experimental condition. **p- value < 0.01; ***p-value < 0.001; ****p-value < 0.0001.


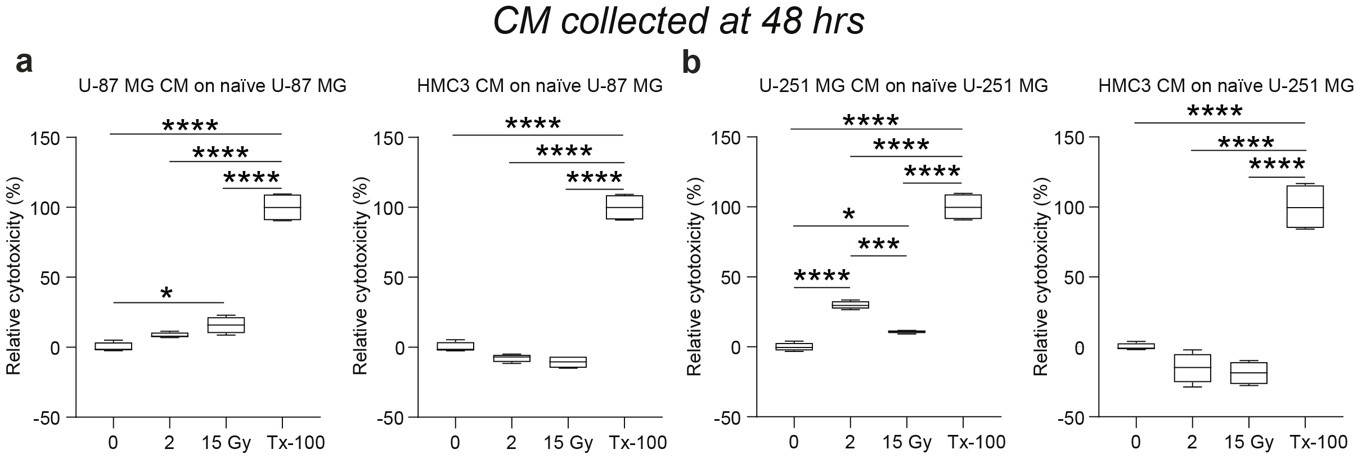


**Supplementary Figure 4.** LDH assay on U-87 MG and U-251 MG cells exposed to homocellular or heterocellular CM collected at 48 hours post-irradiation. a) LDH viability assay on U-87 MG cultures treated with 0 Gy, 2 Gy or 15 Gy U-87 MG CM or HMC3 CM, collected at 48 hours post-IR. b) LDH viability assay on U-251 MG cultures treated with 0 Gy, 2 Gy or 15 Gy U-251 MG CM or HMC3 CM, collected at 48 hours post-IR. Data are shown via standard box and whiskers of n ≥ 4 independent replicates for each experimental condition.*p-value < 0.05; ***p-value < 0.001; ****p-value < 0.0001.


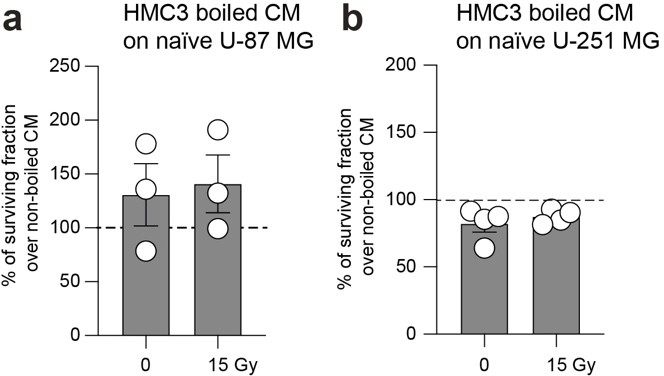


**Supplementary Figure 5. Boiled HMC3 CM retains its effects on U-87 MG and U-251 MG cell lines.** a-b) Surviving fraction and representative pictures of U-87 MG and U-251 MG cell lines exposed to 0 Gy and 15 Gy boiled HMC3 CM. Data are expressed via scattered dot-plot and mean ± SEM of n

≥ 3 independent replicates for each experimental condition.


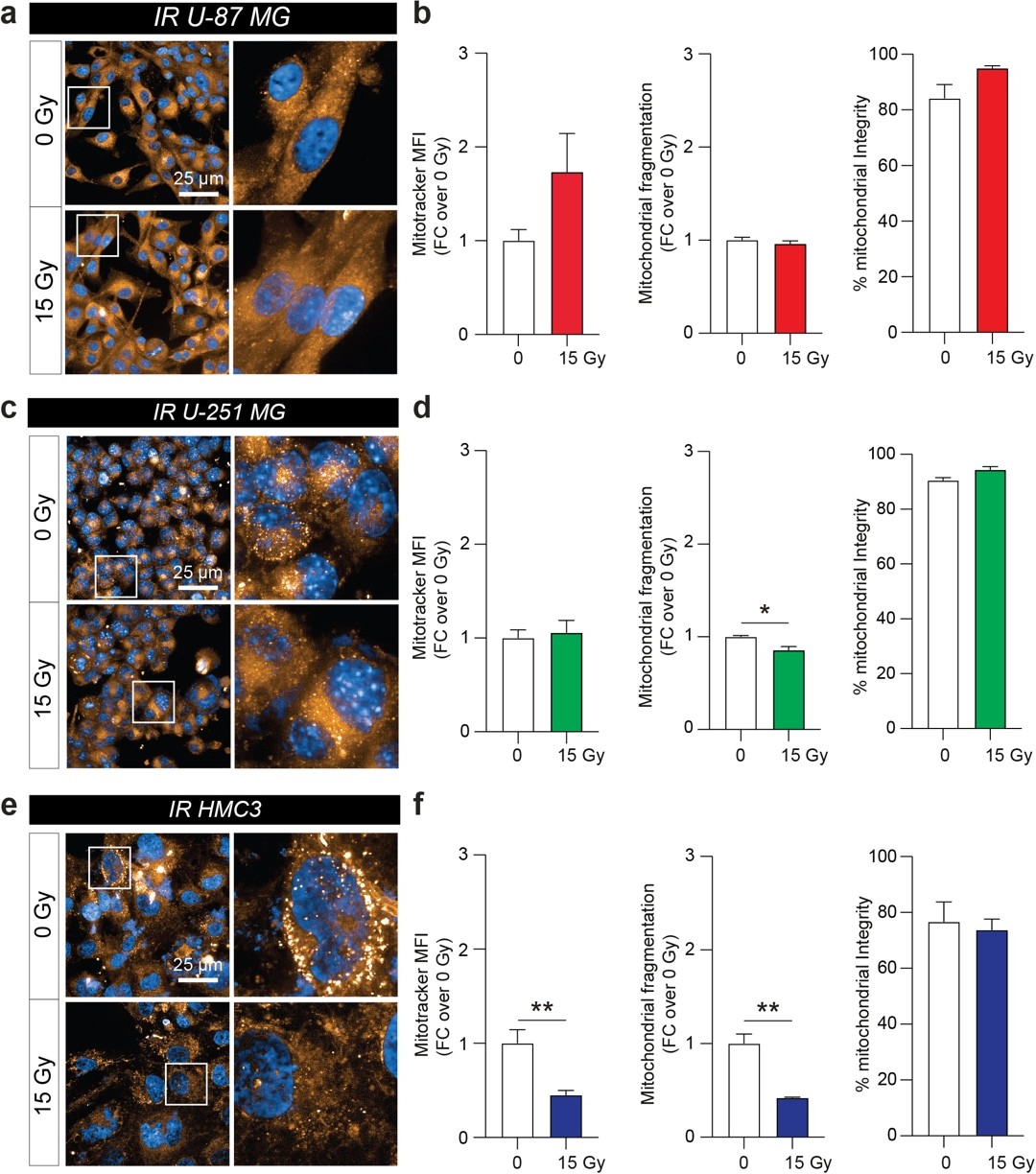


**Supplementary Figure 6. Effects of direct radiation on GBM and microglia mitochondrial fitness.** a-b) Representative pictures of immunofluorescence staining with Mitotracker of 0 Gy and 15 Gy irradiated U-87 MG (a) and high-content analysis of Mitotracker MFI, mitochondrial fragmentation and percentage of mitochondrial integrity (b). c-d) Representative pictures of immunofluorescence staining with Mitotracker of 0 Gy and 15 Gy irradiated U-251 MG (c) and high-content analysis of Mitotracker MFI, mitochondrial fragmentation and percentage of mitochondrial integrity (d). e-f) Representative pictures of immunofluorescence staining with Mitotracker of 0 Gy and 15 Gy irradiated HMC3 (e) and high-content analysis of Mitotracker MFI, mitochondrial fragmentation and percentage of mitochondrial integrity (f). Data are shown as bar plot and expressed as mean ± SEM of n = 4 independent replicates. *p-value < 0.05; **p-value < 0.01.

| **Gene of interest** | **Forward primer (5’ -> 3’)** | **Reverse primer (5’-> 3’)** |
| --- | --- | --- |
| DNM1L | TGGGCGCCGACATCA | GCTCTGCGTTCCCACTACGA |
| FIS1 | AAGAAAGATGGACTCGTGGGC | CCGCGTCTCCTTCAGGATTT |
| MNF1 | ATGCAGTGGGAGTCCGAGC | CAGGGACATTGCGCTTCAC |
| OPA1 | AGGAGCTCATCTGTTTGGAGTC | GCTCACCAAGCAGACCCTTT |
| MNF2 | GCGGAGACTCATAATGGCAGA | TCCGAGATAGCACCTCACCA |
| CYTB | ACGAGCCACCGAAACAGAAT | ACGATTTTCGCCAGTCACCT |
| ND4 | CCAGTGGAATGCCTTGCCTA | TTGATCGCGGTGAGATTCCC |
| TFAM | CCGAGGTGGTTTTCATCTGT | AGTCTTCAGCTTTTCCTGCG |
| ATP5F1A | CCGCCTTCCGCGGTATAATC | ATGTACGCGGGCAATACCAT |
| ACTB | CCTTTGCCGATCCGCCG | AACATGATCTGGGTCATCTTCTCGC |

**Supplementary Table 1.** List of primers’ sequences.
